# Supplementary material for: Hydrodynamic assembly of two-dimensional layered double hydroxide nanostructures
Source: Nat Commun. 2018 Nov 21;9:4913. doi: 10.1038/s41467-018-07395-4 (PMC6249219; doi:10.1038/s41467-018-07395-4)
Supplement: Supplementary file 1 — Supplementary Information [file 41467_2018_7395_MOESM1_ESM.pdf]

Supplementary Information

Hydrodynamic assembly of two-dimensional layered double hydroxide nanostructures

Jose *et al.*

Supplementary Figures

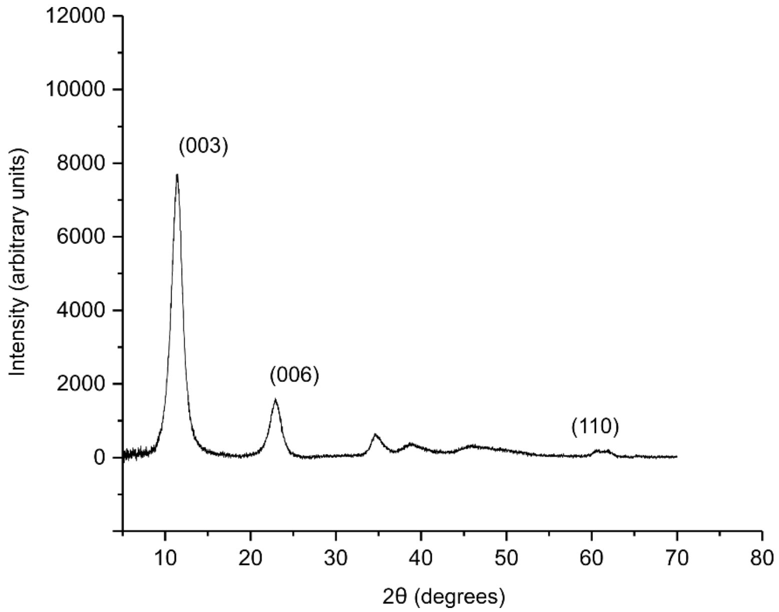

Supplementary Fig. 1 Powder XRD spectra, with characteristic hydrotalcite peaks at 2θ = 11.5°, 22.9°, and 61.8°, corresponding to the (003), (006), and (110) reflections.

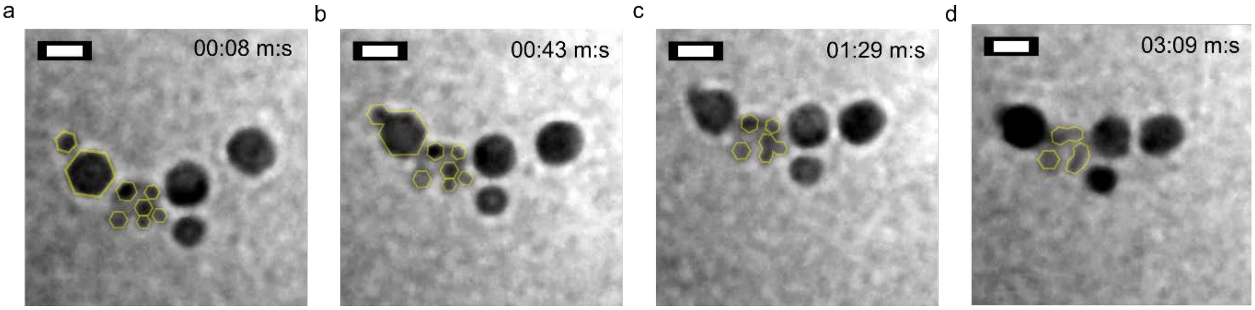

Supplementary Fig. 2 LTEM frames showing the assembly and oriented attachment of ~5 nm particles in a pentagonal configuration. At the start of imaging (a) 43 seconds into imaging the two far left particles attach (b). After about 1.5 minutes three particles in the pentagonal configuration attach (c). After 3 minutes of imaging (d) two other particles in the pentagonal configuration attach, resulting in three closely spaced but unattached particles. Outlines are to guide the eye. Scalebars are 15 nm.

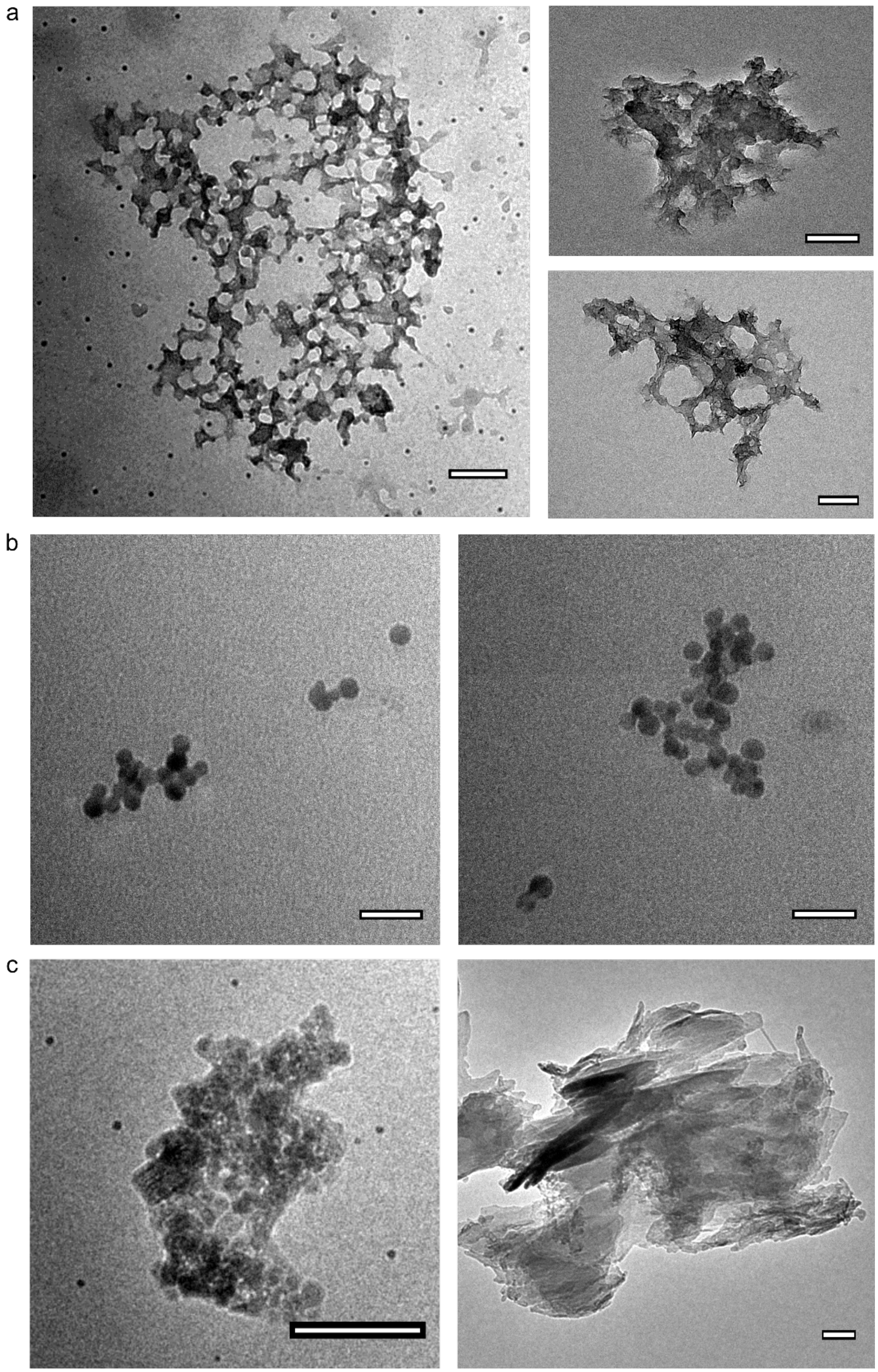

Supplementary Fig. 3 TEM and LTEM images of various aggregates. (a) Fractal aggregates in static LTEM (left) and normal TEM on a carbon film (upper and lower right). Scalebar = 100 nm. (b) 2D Fractal aggregates formed in flow LTEM (left and right). Scalebar = 25 nm. (c) Dense crystalline aggregates in static LTEM (left) and normal TEM (right). Scalebar = 100 nm.

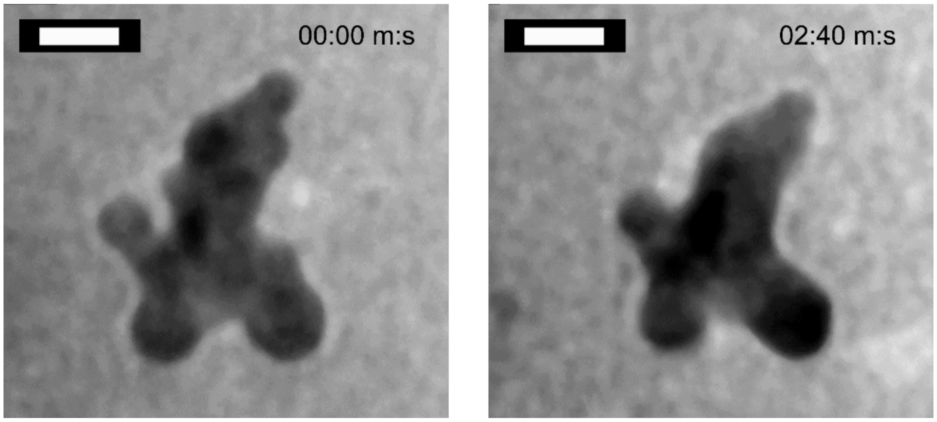

Supplementary Fig. 4 LTEM images of an aggregate annealing over time. Initial aggregate structure (left) at the start of imaging, and the final aggregate structure (right) 2.3 minutes after imaging. Scalebar = 10 nm.

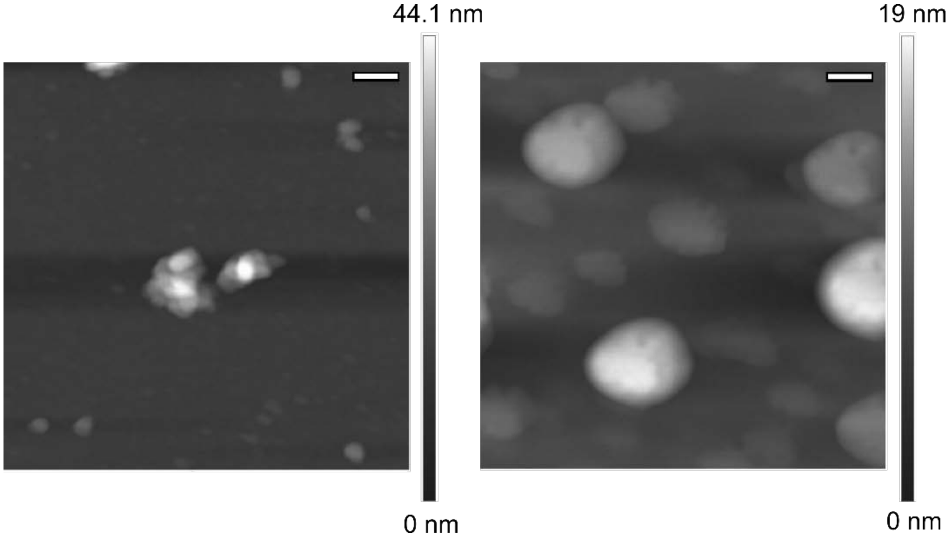

Supplementary Fig. 5 AFM of over-sonicated LDH. AFM height maps of LDH before (left) and after 30 minutes of sonication (right). Scalebar = 100 nm.

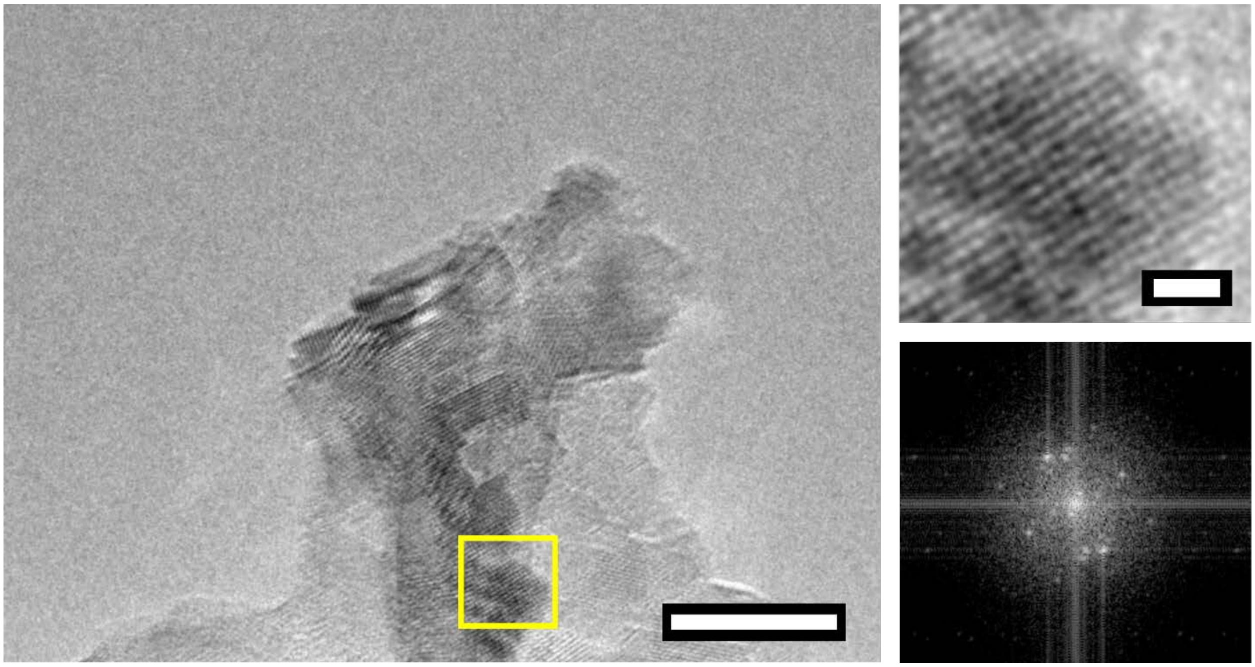

Supplementary Fig. 6 HRTEM of LDH after over-irradiation. HRTEM image (left) of over-irradiated LDH, exhibiting cubic crystallinity following LDH decomposition in the outlined section (upper right), with the corresponding Fourier transform (lower right). Scalebars are 10 nm (left) and 1 nm (upper right).

Supplementary Tables

Supplementary Table 1 Characteristic mixing times for reported geometries.

	Mixing time	Author
Turbulent tangential micromixer	160 ns	Mitic *et al.*(2015) [1]
Capillary-ball micromixer	10 µs	Shastry *et al*. (1998) [2]
Annular microreactor	288 µs	
Impinging jet mixer	1 ms	Baber *et al.* (2016) [3]
Coaxial turbulent jet mixer	10 ms	Lim *et al*. (2014) [4]
Spinning disk reactor	12 ms	Boodhoo *et al.* (2011) [5]
Three phase microreactor	150 ms	Wong *et al.* (2017) [6]
Cylindrical flask, 6 blade turbine	800 ms	Hartman *et al.* (2011) [7]
Cylindrical flask, magnetic stir bar	9.3 s	Hartman *et al.* (2011) [7]

Supplementary Discussion

Aggregate Formation

Aggregates observed in LTEM and HRTEM were either fractal or dense crystalline structures. The fractal structures, as reported in Supplementary Figure 3a, consisted of LDH nanoparticles attached edge-to-edge and face-to-edge. Twisting, branching, and cross-linking created petal-like morphologies, which varied in density. Aggregates formed via bubble-induced flow possessed 2D fractal structures, attached at hexagonal facets, as shown in Supplementary Figure 3b. This leads us to speculate that formation of 3D fractal aggregates could be accelerated by flow, because shear

forces are known to influence the formation of structures ranging from dense crystalline structures with long-range order to highly fractal aggregates[8-11].

The aggregates with higher density, as seen in Supplementary Figure 3c, consisted of highly oriented particles and may be formed via the restructuring of these fractal aggregates. Such restructuring may be in the form of a diffusion-driven annealing process, which was observed in LTEM (see Supplementary Figure 4), or through the physical reorganization of nano-platelets, which has been previously suggested by[12]. Due to the high tendency of these particles to agglomerate, reorganization may be a kinetically limited process that requires time and input of energy, which may be the reason why many LDH co-precipitation protocols require a hydrothermal aging step to achieve high crystallinity[13].

The ability to tailor the fractal and crystalline structures of LDH and 2D materials in general is important in catalytic applications, where functionality as a catalyst and catalyst support is highly dependent on surface area and morphology. Such structuring is difficult with nano-LDH. [14] noted that the synthesis of nano-LDH results in highly dense structures with low surface area, and, in fact, most flow synthesis protocols create crystalline/disperse structures[15-17]. High surface area, flower-like LDH structures, are often synthesized in solvothermal processes[18-20].

Here we see that high shear rates may be used to generate unique fractal structures with anisotropic nanomaterials; however, hydrodynamic control is not often applied in practice. The use of hydrodynamics instead of solvothermal methods could lead to cleaner processes for manufacturing of nanomaterials. The formation and reorganization of such structures under shear is worth considering in future studies, with the effect of size, polydispersity, shear rate, particle density, and residence time.

Gradient Tensor Decomposition

The velocity field in this case is similar to simple shear flow. The analytical solution for the annular flow velocity distribution in two-dimensional coordinates, assuming that flow is axisymmetric, smooth and laminar, is given in Supplementary Equations 1 and 2, where u_1 and u_2 are the velocities in the axial and radial directions (m s^{-1}), x_1 is the axial distance, x_2 is the radial distance from the tube axis (m), μ is the liquid viscosity (Pa s) and B is the axial pressure gradient (Pa m^{-1})[21].

$$u_1 = -\frac{BR^2}{4\mu}\left[1-\left(\frac{x_2}{R}\right)^2\right] \quad (1)$$

$$u_2 = 0 \quad (2)$$

Decomposing the gradient tensor $\partial u_i/\partial x_j$ into the rate-of-strain tensor $\mathbf{S}$ nd rate-of-rotation tensor $\mathbf{R}$ with Supplementary Equations 3 - 5 yields the tensors in Supplementary Equations 6 and 7.

$$\frac{\partial u_i}{\partial x_j} = \mathbf{S}_{\boldsymbol{ij}} + \mathbf{R}_{\boldsymbol{ij}} \quad (3)$$

$$\mathbf{S}_{\boldsymbol{ij}} = \frac{1}{2}\left(\frac{\partial u_i}{\partial x_j} + \frac{\partial u_j}{\partial x_i}\right) \quad (4)$$

$$\mathbf{R}_{\boldsymbol{ij}} = \frac{1}{2}\left(\frac{\partial u_i}{\partial x_j} - \frac{\partial u_j}{\partial x_i}\right) \quad (5)$$

$$\mathbf{S} = \begin{pmatrix} 0 & \frac{BR^2}{2\mu}x_2 \\ \frac{BR^2}{2\mu}x_2 & 0 \end{pmatrix} \quad 6$$

$$\mathbf{R} = \begin{pmatrix} 0 & \frac{BR^2}{2\mu}x_2 \\ -\frac{BR^2}{2\mu}x_2 & 0 \end{pmatrix} \quad 7$$

For a thin liquid layer (a small range of R) the rate of strain and rotational component are relatively constant over the volume of the liquid, compared to other flow regimes like pipe flow or Taylor flow. For this reason, the effects on aggregation are similar to that of simple shear flow, which are well studied for hard spheres and are currently areas of investigation for soft matter and complex-shaped particles.

For anisotropic particles, at shear rates at the order of $\mathrm{Pe_r} \sim 1$ the rotational component will help control the orientation of anisotropic particles in flow. The orbits of disc-like particles about their axis will slow and become periodic, with becoming slowest when oriented close to the flow gradient direction[22]. The pair-interaction distribution will be inhomogeneous, such that interactions along compressive axis are favored and convected away along the extensional axis[23]. By orienting particles relative to one another oriented aggregation is more favorable.

While shear may accelerate aggregation, above a certain threshold, stresses on aggregates will be high enough to cause breakup. Shear stress exerted by simple shear flow will cause breakup along the extensional axis and aggregate along the compressive axis, which may cause aggregate anisotropy[24]. This would further enhance the anisotropy of disc aggregates, due to their orientation in flow. This is why high shear rates used in this study resulted in thin aggregates.

At certain flowrates Kelvin-Helmholtz instabilities will occur, introducing deviations from the smooth, laminar analytical solution. Turbulence created by instabilities will cause deviations in particle velocities and trajectories from their behavior in simple shear. For this reason numerical simulations and experimental observation are more reliable in determining flow characteristics, and predicting the occurrence of instabilities.

Calculation of Growth Rate using the Sherwood Number

We have used the Smoluchowski equation to directly provide a growth rate in terms of mol/s from a first principles approach, which is also similar to the approach used to calculate growth rates via aggregation under shear. If we calculate the growth rate using the semi-empirical approach using the Sherwood number (Sh) we still obtain a similar result for the growth rate. See calculations below.

To use the Sherwood approach, we consider a hexagonal particle with side-length (a = 2 nm), which we use as the characteristic length scale. The Sherwood number is expressed with the correlation in Supplementary Equation 8[25], where k_d is the mass transfer coefficient, D_A is the diffusion coefficient of solute species A ($1.02 \cdot 10^{-9}$ m^{2}s^{-1}), $\mathrm{Re} = u_s a\rho/\mu$ is Reynolds number (u_s is the particle slip velocity, ρ is the solution density, μ is the solution dynamic viscosity) and $\mathrm{Sc} = \mu/\rho D_\mathrm{A}$ is the Schmidt number. For very small particles (<10 nm) the slip velocity of the particle (u_s), such that $\mathrm{Sh} \approx 2$.

$$\mathrm{Sh} = \frac{k_\mathrm{d} 2a}{D_\mathrm{A}} = 2 + 1.10\mathrm{Re}^{\frac{1}{2}}\mathrm{Sc}^{\frac{1}{3}} \approx 2 \quad 8$$

We can then use the semiempirical relation in Supplementary Equation 9 to obtain G, which is the growth rate of the particle along its characteristic growth dimension in m s^{-1}, where ρ_c is the crystal density (2.05·10^3 mol m^{-3}) and we assume the difference between the initial bulk concentration of A ($[A]_0$=0.75·10^{-3} mol m^{-3}) is much greater than the crystal-surface interface concentration of A ($[A]_i$).

$$G = \frac{2k_\mathrm{d}}{\rho_\mathrm{c}}([A]_0 - [A]_i) \approx \frac{2D_\mathrm{A}}{\rho_\mathrm{c}a}[A]_0 \qquad 9$$

To then convert G into $r_{\mathrm{g},n}$, the growth rate of a particle of size n along a (mol s^{-1}), we use Supplementary Equation 10, which converts the growth rate into the mass growth rate of hexagonal particles, where H is the hexagonal particle thickness (0.7 nm), N_A is Avogadro's constant (6.02·10^{23} mol^{-1}) and $[B_n]$ is the concentration of particles of size n (2·10^{-7} mol m^{-3}).

$$r_{\mathrm{g},n} = 3\sqrt{3}HaG\rho_\mathrm{c}N_\mathrm{A}[B_n] \qquad 10$$

Inserting $r_{\mathrm{g},n}$ into Supplementary Equation 11, we solve for the kinetic growth rate constant K_n^g, which is very similar to the previously calculated 1.42·10^7 $m^3s^{-1}mol^{-1}$, considering the approximations involved.

$$K_n^\mathrm{g} = \frac{r_{\mathrm{g},n}}{[A][B_n]} = 4.5 \cdot 10^6 \ \mathrm{m^3s^{-1}mol^{-1}} \qquad 11$$

Supplementary References

1 Mitic, S., van Nieuwkasteele, A. W., van den Berg, A. & de Vries, S. Design of turbulent tangential micro-mixers that mix liquids on the nanosecond time scale. *Anal Biochem* **469**, 19-26, doi:10.1016/j.ab.2014.10.003 (2015).

2 Shastry, M. C. R., Luck, S. D. & Roder, H. A continuous-flow capillary mixing method to monitor reactions on the microsecond time scale. *Biophysical Journal* **74**, 2714-2721 (1998).

3 Baber, R., Mazzei, L., Thanh, N. T. K. & Gavriilidis, A. Synthesis of Silver Nanoparticles Using a Microfluidic Impinging Jet Reactor. *J Flow Chem* **6**, 268-278, doi:10.1556/1846.2016.00015 (2016).

4 Lim, J. M. *et al.* Ultra-High Throughput Synthesis of Nanoparticles with Homogeneous Size Distribution Using a Coaxial Turbulent Jet Mixer. *Acs Nano* **8**, 6056-6065, doi:10.1021/nn501371n (2014).

5 Boodhoo, K. V. K. & Al-Hengari, S. R. Micromixing Characteristics in a Small-Scale Spinning Disk Reactor Chemical Engineering & Technology Volume 35, Issue 7. *Chemical Engineering & Technology* **35**, 1229-1237 (2012). <http://onlinelibrary.wiley.com/doi/10.1002/ceat.201100695/abstract>.

6 Wong, W. K. *et al.* Robust, non-fouling liters-per-day flow synthesis of ultra-small catalytically active metal nanoparticles in a single-channel reactor. *React Chem Eng* **2**, 636-641, doi:10.1039/C7RE00072C (2017).

7 Hartman, R. L., McMullen, J. P. & Jensen, K. F. Deciding Whether To Go with the Flow: Evaluating the Merits of Flow Reactors for Synthesis. *Angewandte Chemie-International Edition* **50**, 7502-7519, doi:10.1002/anie.201004637 (2011).

8 Conchuir, B. O., Harshe, Y. M., Lattuada, M. & Zaccone, A. Analytical Model of Fractal Aggregate Stability and Restructuring in Shear Flows. *Ind Eng Chem Res* **53**, 9109-9119, doi:10.1021/ie4032605 (2014).

9 Ackerson, B. J. Shear Induced Order of Hard-Sphere Suspensions. *J Phys-Condens Mat* **2**, Sa389-Sa392 (1990).

10 Chen, L. B., Chow, M. K., Ackerson, B. J. & Zukoski, C. F. Rheological and Microstructural Transitions in Colloidal Crystals. *Langmuir* **10**, 2817-2829, doi:DOI 10.1021/la00020a052 (1994).
11 Hanley, H. J. M. *et al.* Shear-induced restructuring of concentrated colloidal silica gels. *J Phys-Condens Mat* **11**, 1369-1380, doi:Doi 10.1088/0953-8984/11/6/003 (1999).
12 Pang, X. J. *et al.* Growth behavior of water dispersed MgAl layered double hydroxide nanosheets. *Rsc Advances* **7**, 14989-14997, doi:10.1039/c7ra00833c (2017).
13 Cavani, F., Trifiro, F. & Vaccari, A. Hydrotalcite-Type Anionic Clays: Preparation, Properties and Applications. *Catal Today* **11**, 173-301, doi:Doi 10.1016/0920-5861(91)80068-K (1991).
14 Abello, S., Mitchell, S., Santiago, M., Stoica, G. & Perez-Ramirez, J. Perturbing the properties of layered double hydroxides by continuous coprecipitation with short residence time. *Journal of Materials Chemistry* **20**, 5878-5887, doi:10.1039/c0jm00088d (2010).
15 Wang, Q., Tang, S. V. Y., Lester, E. & O'Hare, D. Synthesis of ultrafine layered double hydroxide (LDHs) nanoplates using a continuous-flow hydrothermal reactor. *Nanoscale* **5**, 114-117, doi:10.1039/c2nr32568c (2013).
16 Pang, X. J., Sun, M. Y., Ma, X. M. & Hou, W. G. Synthesis of layered double hydroxide nanosheets by coprecipitation using a T-type microchannel reactor. *J Solid State Chem* **210**, 111-115, doi:10.1016/j.jssc.2013.11.013 (2014).
17 Ren, M. Y., Yang, M., Chen, G. W. & Yuan, Q. High-Throughput Preparation of Monodispersed Layered Double Hydroxides via Microreaction Technology. *J Flow Chem* **4**, 164-167, doi:10.1556/Jfc-D-14-00014 (2014).
18 Li, P. & Zeng, H. C. Immobilization of Metal-Organic Framework Nanocrystals for Advanced Design of Supported Nanocatalysts. *Acs Appl Mater Inter* **8**, 29551-29564, doi:10.1021/acsami.6b11775 (2016).
19 Zhang, F. Z., Zhang, Y., Yue, C. L., Zhang, R. & Yang, Y. M. Facile Fabrication of Spherical Architecture of Ni/Al Layered Double Hydroxide Based on In Situ Transformation Mechanism. *Aiche J* **60**, 4027-4036, doi:10.1002/aic.14609 (2014).
20 Lv, W. Y., Du, M., Ye, W. J. & Zheng, Q. The formation mechanism of layered double hydroxide nanoscrolls by facile trinal-phase hydrothermal treatment and their adsorption properties. *Journal of Materials Chemistry A* **3**, 23395-23402, doi:10.1039/c5ta05218a (2015).
21 Guo, Z., Fletcher, D. F. & Haynes, B. S. Numerical simulation of annular flow hydrodynamics in microchannels. *Computers & Fluids* **133**, 90-102, doi:https://doi.org/10.1016/j.compfluid.2016.04.017 (2016).
22 Mewis, J. & Wagner, N. J. *Colloidal Suspension Rheology*. (Cambridge University Press, 2012).
23 Foss, D. R. & Brady, J. F. Structure, diffusion and rheology of Brownian suspensions by Stokesian Dynamics simulation. *J Fluid Mech* **407**, 167-200, doi:10.1017/s0022112099007557 (2000).
24 Hoekstra, H., Vermant, J., Mewis, J. & Fuller, G. G. Flow-Induced Anisotropy and Reversible Aggregation in Two-Dimensional Suspensions. *Langmuir* **19**, 9134-9141, doi:10.1021/la034582k (2003).
25 Frossling, N. Uber die Verdunstung fallender Tropfen. *Beitr. Geophys. Gerlands* **52**, 170-216 (1938).
