# Supplementary material for: Hydrodynamic assembly of two-dimensional layered double hydroxide nanostructures
Source: Nat Commun. 2018 Nov 21;9:4913. doi: 10.1038/s41467-018-07395-4 (PMC6249219; doi:10.1038/s41467-018-07395-4)
Supplement: Supplementary file 3 — Description of Additional Supplementary Information [file 41467_2018_7395_MOESM3_ESM.pdf]

Description of Additional Supplementary Files

File Name: Supplementary Movie 1
Description: Light-induced fluorescence microscopy movie of reactor mixing dynamics, viewing the axial cross-section. Dimensions are given in micrometers (um). Phase 1 (top stream, red, laced with fluorescent tracer) and Phase 2 (bottom stream, blank) flow from left to right. At top is half of the axial cross-section, where T2 and T3 are highlighted, showing chaotic mixing over 1 mm.

File Name: Supplementary Movie 2
Description: Liquid transmission electron microscopy movie of layered double hydroxide nanoplatelets aggregating via oriented attachment. Scalebar is 10 nm. Time elapsed is given in minutes:seconds (m:s).

File Name: Supplementary Movie 3
Description: Liquid transmission electron microscopy movie of layered double hydroxide nanoplatelets attaching in different configurations. Scalebar is 15 nm. Time elapsed is given in minutes:seconds (m:s).

File Name: Supplementary Movie 4
Description: Liquid transmission electron microscopy movie of two layered double hydroxide nanoplatelets attaching under static conditions. Particles and the interparticle distance are outlined in blue. Scalebar is 10 nm. Time elapsed is given in minutes:seconds (m:s).

File Name: Supplementary Movie 5
Description: Liquid transmission electron microscopy movie of two layered double hydroxide nanoplatelets attaching under flow conditions. Particles and the interparticle distance are outlined in red. Scalebar is 20 nm. Time elapsed is given in minutes:seconds (m:s).
